# Effects of Rainfall Exclusion Treatment on Photosynthetic Characteristics of Black Locust in the Sub-Humid Region of the Loess Plateau, China

**DOI:** 10.3390/plants13050704

**Published:** 2024-03-01

**Authors:** Haining Guo, Yiran Wang, Guoqing Li, Sheng Du

**Affiliations:** 1 State Key Laboratory of Soil Erosion and Dryland Farming on the Loess Plateau, Northwest A&F University, Yangling 712100, Shaanxi, China; bestghn@163.com (H.G.); m15504215781@163.com (Y.W.); liguoqing@nwsuaf.edu.cn (G.L.); 2 College of Forestry, Northwest A&F University, Yangling 712100, Shaanxi, China; 3 Institute of Soil and Water Conservation, Chinese Academy of Sciences and Ministry of Water Resources, Yangling 712100, Shaanxi, China

**Keywords:** black locust, *Robinia pseudoacacia*, drought, photosynthesis, leaf gas exchange, light response curve, CO_2_ response curve

## Abstract

The mesic-origin species *Robinia pseudoacacia* L. (black locust) is widely planted in the semiarid and sub-humid areas of the Loess Plateau for the reforestation of vegetation-degraded land. Under the scenario of changing precipitation patterns, exploring the response of photosynthesis to drought allows us to assess the risk to sustainable development of these plantations. In this study, paired plots were established including the control and a treatment of 30% exclusion of throughfall (since 2018). The photosynthetic characteristics were investigated using a portable photosynthesis system for four periods in the full-leaf growing season of 2021–2022, the fourth and fifth years, on both treated and controlled sampling trees. Leaf gas exchange parameters derived from diurnal changing patterns, light response curves, and CO_2_ response curves showed significant differences except for period II (9–11 September 2021) between the two plots. The photosynthetic midday depression was observed in 2022 in the treated plot. Meanwhile, the decline of net photosynthetic rate in the treated plot was converted from stomatal limitation to non-stomatal limitation. Furthermore, we observed that black locust adapted to long-term water deficiency by reducing stomatal conductance, increasing water use efficiency and intrinsic water use efficiency. The results demonstrate that reduction in precipitation would cause photosynthesis decrease, weaken the response sensitivity to light and CO_2_, and potentially impair photosynthetic resilience of the plantations. They also provide insights into the changes in photosynthetic functions under global climate change and a reference for management of plantations.

## 1. Introduction

Photosynthesis is the physiological basis for the growth and yield of trees, as well as the maintenance of the global carbon cycle and ecological balance in nature [1]. Drought has a negative effect on photosynthesis in many species [2,3,4,5]. For example, short-term drought reduced net assimilation rate of species, and photosynthesis was primarily limited by stomatal conductance [6]. Temporary drought significantly decreased light saturation point, altered the diurnal changes of gas exchange, and increased intrinsic water use efficiency [7]. Plant responses to drought are diverse and depend on species and rate of progression of water stress [8,9]. Vaz et al. [10] found that stomatal conductance and photosynthetic rate peaked in spring, progressively declined during seasonal drought throughout summer, and recovered well after autumn rainfall. The photosynthetic limitation under mild drought was dominated by stomatal conductance, whereas non-stomatal limitation occurred under moderate and severe drought [11]. The permanent water deficit increased the intrinsic water use efficiency of trees, resulting in a bimodal diurnal variation of photosynthetic rate, transpiration rate, and stomatal conductance with a clear midday depression [12]. Knowledge about the response of photosynthesis to drought is accumulating; however, the response is variable, and even within a species, different genotypes respond differently.

Global climate change has affected photosynthesis in plantations worldwide to varying degrees [13]. Emissions of greenhouse gases have accelerated global climate change, increased vapor pressure deficit, and changed regional precipitation patterns [14]. These changes will trigger increases in drought duration and intensity [15,16], leading to water deficit in forest ecosystems [17] and directly affecting photosynthesis of trees. In recent years, some researchers have tended to use artificial rainfall exclusion treatment to simulate the changes in regional rainfall patterns and elucidate the effects of water deficit on tree photosynthesis [18,19,20]. For instance, seasonal drought and throughfall exclusion significantly decreased daily whole-crown photosynthesis, transpiration, and stomatal conductance [21]. The rainfall exclusion increased the relative importance of non-stomatal limitation with increasing drought duration and intensity in the *Quercus ilex* ecosystem [22]. Limousin et al. [23] demonstrated that rainfall exclusion significantly reduced light-saturated net assimilation rate, carboxylation rate, maximum rate of electron transport, and weakened the sensitivity to environmental factors. Under long-term rainfall exclusion treatment, the decline in photosynthesis of the treated plot was similar to that of the control plot during seasonal drought, but sufficient late-season rainfalls were not enough to restore photosynthesis in the treated plot to early-summer values [24]. Our study adopted this method to investigate the effects of drought on plantation photosynthesis in the Loess Plateau region of China.

The Loess Plateau in China is a major area where soil and water conservation and vegetation restoration are performed due to serious land degradation and soil erosion. Owing to its efficient role in soil conservation and high tolerance to drought, black locust has been introduced as a dominant afforestation species on vegetation-degraded land of the Loess Plateau [25]. The climate of this region presents a trend of drying and warming [26], with a decrease in the regionally averaged rainfall intensity and an increase in consecutive dry days [27]. Rainfall is the only source of soil water supplement in this region [28]. This will inevitably aggravate the contradiction between black locust and water resources, affecting plantation photosynthesis and productivity. Thus, understanding the responses of black locust photosynthesis to drought in this region will enable us to evaluate the sustainability of vegetation under future climate change.

The black locust has developed some morphological features and anatomical adaptations to conduct photosynthesis under water deficit, such as low stomatal density, thick bark, and spines [29,30,31,32]. On the other hand, black locust has not been clearly defined as drought-sensitive or drought-tolerant. Du et al. [33] reported that the exotic black locust was drought-sensitive in the semiarid Loess Plateau region. He et al. [30] found that this species has physiological responses of strong stomatal control to drought in the sub-humid region of the Loess Plateau. However, some clones of black locust can tolerate water stress and were considered as drought-tolerant type in Napkor [34]. The black locust showed relatively lower sensitivity of stomatal conductance to environmental factors in a semiarid site [35]. Different water use strategies can alter the stomatal conductance of this species, which in turn affects photosynthesis. Therefore, the response and adaptation of black locust photosynthesis to rainfall changes and how it avoids hydraulic imbalance and carbon starvation need to be further studied.

Using the platform of a rainfall exclusion experiment for a black locust plantation in a sub-humid site of the Loess Plateau, this study analyzed the photosynthetic performance and associated parameters in four periods with potential variation of soil water content. Our main objectives were to (i) compare the relevant photosynthetic parameters between the two plots, (ii) quantitatively determine whether the sensitivities of net photosynthetic rate to light intensity and CO_2_ concentration varies with soil water content, and (iii) explore the mechanism of photosynthesis response to soil moisture changes under the rainfall exclusion experiment. The results could also provide a practical basis for sustainable development and plantation management of black locust in the region.

## 2. Materials and Methods

### 2.1. Study Site and Rainfall Exclusion Treatment

This study was performed in 2021–2022 at the platform of a rainfall exclusion experiment for a black locust plantation in Huaiping forest station of Yongshou county, Shaanxi Province of China (34°80′ N, 107°97′ E, 1430 m a.s.l.). The site belongs to the sub-humid area of the southern Loess Plateau, with the growing season for usual deciduous species extending from April to October. The annual mean precipitation and annual mean temperature recorded by a meteorological station 20 km away in the town (1966–2005) was 620 mm and 10.8 °C, respectively, with 70% of precipitation being distributed from June to September.

The black locust plantation at the study site was around 18 years old in 2021, with the mean diameter at breast height of 9.3 cm and the mean tree height of 10.4 m. Undergrowth vegetation includes a few shrubs of *Rubus idaeus*, *Cornus alba*, and *Spiraea salicifolia*, and grasses of *Elymus kamoji*, *Festuca ovina*, and *Rubia cordifolia*. A 20 m × 50 m large plot was established in 2018, which was divided into two plots (10 m × 50 m) and marked as the control and rainfall exclusion treatment plots. In order to measure photosynthesis of canopy leaves, a frame of stainless steel was constructed to the canopy height (12 m) to measure the gas exchange parameters of leaves. Three representative trees in each plot were studied from 2021 to 2022.

To investigate the response of photosynthesis of black locust plantations to changed regional precipitation patterns in the sub-humid region, a rainfall exclusion treatment was started in March 2018 by setting transparent waterproof panels (1.5 m in height) between the rows of trees to divert part of the throughfall outside the plot. According to the proportion of panels’ area, 30% of the throughfall was excluded. The rainfall of the treated plot was approximately equal to that of the semiarid region of the Loess Plateau, China (Yan’an). To prevent soil moisture exchange between inside and outside the treated plot, aluminum-plastic sheets were inserted vertically into the ground around the treated plot, with 80 cm being underground and 20 cm left above.

### 2.2. Diurnal Course Measurement of Canopy Leaf Gas Exchange

Leaf net photosynthetic rate (Pn, μmol·m^−2^·s^−1^), stomatal conductance (gs, mol m^−2^·s^−1^), intercellular CO_2_ concentration (Ci, μmol mol^−1^), transpiration rate (Tr, mmol·m^−2^·s^−1^), photosynthetically active radiation (*PAR*, μmol·m^−2^·s^−1^), and vapor pressure deficit (*VPD*, kPa) were measured by a portable photosynthesis system analyzer (LI-6400XT, LI-COR, Inc., Lincoln, NE, USA). According to the size and shape of the black locust leaves, this analyzer was equipped with an integrated leaf chamber fluorometer (LI-6400-40, LI-COR, Inc., Lincoln, NE, USA) using a red-blue light source.

The diurnal courses of leaf gas exchange were measured on a sunny day or day with fewer clouds in each period. Measurements were carried out during 6:00–19:00 at 1 h intervals and matched before logging. The *PAR*, CO_2_ concentration, foliar temperature, and air humidity inside the leaf chamber was set to be consistent with the external environment. In each 1 h interval, 3 marked, healthy, mature, and similar-size leaves were measured in each of the three replicate trees in each plot. The stomatal limitation value (Ls) was the ratio of the difference between leaf chamber CO_2_ concentration and intercellular CO_2_ concentration to leaf chamber CO_2_ concentration. The carboxylation efficiency (CE, mol m^−2^ s^−1^) was calculated as the leaf net photosynthetic rate and intercellular CO_2_ concentration ratio. The intrinsic water use efficiency (WUEi, μmol CO_2_/mol H_2_O) was calculated as the leaf net photosynthetic rate and stomatal conductance ratio. The water use efficiency (WUE, μmol CO_2_/mmol H_2_O) was calculated as the leaf net photosynthetic rate and transpiration rate ratio.

### 2.3. Light Response Curve

The response of net photosynthetic rate of six trees to different light intensities were measured between 8:00–11:00 in sunny days. The light response curve of each sample tree was measured twice in each period in 2021 and once in each period in 2022. Before the measurement, the instrument was set to 400 μmol·mol^−1^ CO_2_ with a CO_2_ injection system, air humidity at 60%, flow rate at 500 μmol·s^−1^, *PAR* at 1700 μmol·m^−2^·s^−1^, foliar temperature at 27.0 °C, and the leaves were photoinduced. During the measurement, *PAR* was set by stepping down from 2000 μmol m^−2^ s^−1^ to 1500, 1000, 800, 600, 400, 200, 100, 70, 40, 20, and 0 μmol m^−2^ s^−1^. The minimum and maximum wait times to record data were 120 s and 240 s, respectively. The curve was measured on 1 leaf for each sample tree. The modified rectangular hyperbola model proposed in recent years overcomes the limitations of other models to a certain extent, and can accurately fit and analyze photosynthetic response curve under water deficit condition [36,37]. Hence, modified rectangular hyperbolic model [36] was used to fit the light response curve for the measured data. Light saturation point (LSP, μmol·m^−2^·s^−1^), light compensation point (LCP, μmol·m^−2^·s^−1^), quantum use efficiency (AQE, μmol·μmol^−1^), maximum net photosynthetic rate (P_max_, μmol·m^−2^·s^−1^), and dark respiration rate (R_d_, μmol·m^−2^·s^−1^) were obtained. The model is shown as follows:

(1) $$Pn\left(I\right)=\alpha \frac{1-\beta I}{1+\gamma I}I-R_{d}$$

where Pn is net photosynthetic rate; I is *PAR*; *α* is initial quantum use efficiency of light response curve; *β* and *γ* are adjusting factors; R_d_ is dark respiration rate.

### 2.4. CO_2_ Response Curve

For CO_2_ response curve measurement, the instrument was set as for the light response curve, with the CO_2_ concentrations at 400, 200, 150, 100, 50, 20, 400, 600, 800, 1000, 1500 μmol·mol^−1^ and *PAR* at 1700 μmol·m^−2^·s^−1^. Measurements of six trees were performed between 8:00–11:00 on a sunny day in each period in 2022. The CO_2_ response curves were not measured in 2021. The curve was measured on 1 leaf for each sample tree. The data were also fitted by modified rectangular hyperbolic model [36], and relevant parameters were calculated, including CO_2_ saturation point (CSP, μmol·mol^−1^), CO_2_ compensation point (CCP, μmol·mol^−1^), day respiration rate (R_p_, μmol·m^−2^·s^−1^), maximum net photosynthetic rate (P_max_, μmol·m^−2^·s^−1^), and carboxylation efficiency (CE, mol·m^−2^·s^−1^). The model is shown as follows:

(2) $$Pn\left(Ci\right)=\alpha \frac{1-\beta Ci}{1+\gamma Ci}Ci-R_{p}$$

where Pn is net photosynthetic rate; Ci is intercellular CO_2_ concentration; *α* is initial carboxylation efficiency of CO_2_ response curve; *β* and *γ* are adjusting factors; R_p_ is day respiration rate.

### 2.5. Measurement of Precipitation and Soil Water Content

A weather station was established in an open area outside the stand to record basic meteorological factors. Precipitation was measured by a tipping bucket rain gauge (Model 7852, Davis Ins., Hayward, CA, USA) at approximately 0.5 m above ground. Soil volumetric water content (SWC) of the 0–100 cm soil profile in each plot was monitored with EC-5 sensors (Decagon, Pullman, WA, USA), connecting to a EM50 data logger (Meter, Pullman, WA, USA) and recording 1 h averages. Four sensors were installed at depths of 10, 30, 50 and 90 cm to represent relevant horizons along the whole profile. The weighted average was calculated for the mean SWC of the whole horizon. The records with different weights were averaged according to the relative thickness represented by sensors [38] as follows:

SWC_0–100_ = 0.15 SWC_10_ + 0.2 SWC_30_ + 0.25 SWC_50_ + 0.4 SWC_90_ (3)

where SWC_10_, SWC_30_, SWC_50_, and SWC_90_ are the data measured by sensors at 10, 30, 50 and 90 cm, respectively. The SWC_0–100_ is soil water content of the 0–100 cm profile after weighted averaging.

### 2.6. Statistical Analysis

Significance of differences for relevant photosynthetic parameters and response curve parameters were tested by *t*-test between plots and between measurement periods. The effects of treatment, period, and their interactions on photosynthetic parameters were analyzed by repeated measures ANOVA. To avoid pseudoreplication problems, the three measurements from each tree were averaged before the three replicate trees in each plot were averaged. All data are presented as the means with standard error (*n* = 3). SigmaPlot 14.0 (Systat Software Inc., San Jose, CA, USA) and SPSS 21.0 (IBM Corp., Armonk, NY, USA) were used for graph plotting and statistical analysis, respectively.

## 3. Results

### 3.1. Precipitation and Soil Water Content during the Study Periods

**Figure 1:**
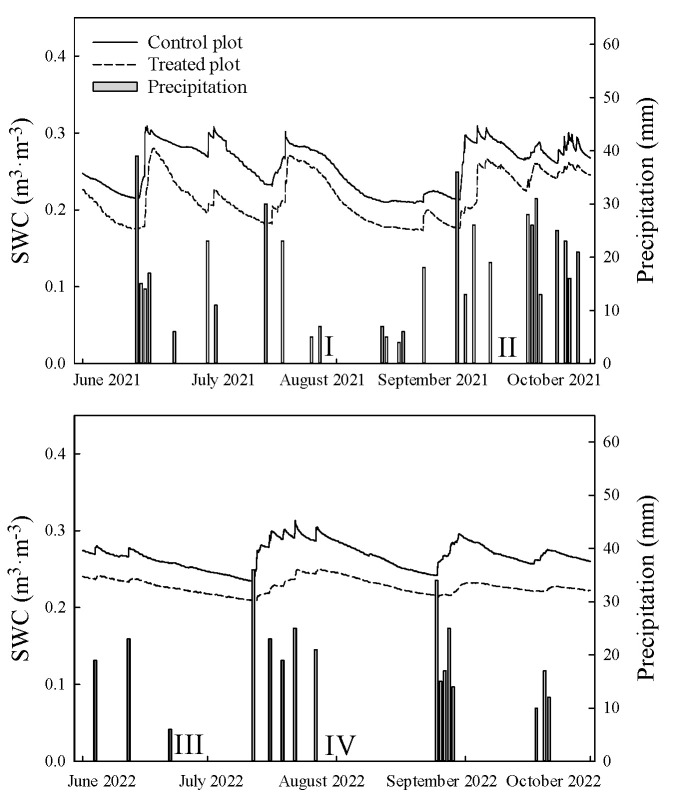
Hourly averages soil water content (SWC, m^3^·m^−3^) in 1 m profile and daily precipitation during the full-leaf growing seasons in the two measurement years. I: period I (28–30 July 2021). II: period II (9–11 September 2021). III: period III (23–25 June 2022). IV: period IV (1–3 August 2022).

Figure 1 shows hourly averages of soil water content in 1 m profile and daily precipitation from June to September in the two study years. SWC in the treated plot was persistently lower than that in the control plot, but the trend of changes was identical throughout the study period. There were more rainfall events from June to September in 2021 than 2022. To investigate the photosynthetic characteristics with respect to SWC fluctuations, we selected four periods (3 sunny days in each) in the vigorous growing season of these two years (the fourth and fifth years of treatment) for the measurements. Period I and II were before and after several rainfall events in 2021, respectively, and could represent the conditions of relatively low and high SWC. Periods III and IV were before and after rainfall in 2022, respectively, and could represent the conditions of relatively low and high SWC (Table 1).

### 3.2. Variations in the Diurnal Courses of Photosynthetic Parameters

**Figure 2:**
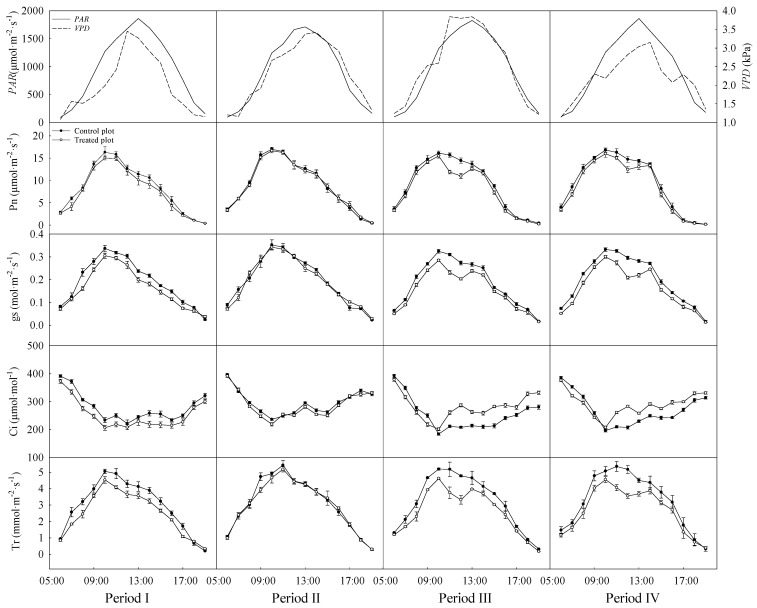
Diurnal courses of photosynthetically active radiation (*PAR*), vapor pressure deficit (*VPD*), net photosynthetic rate (Pn), stomatal conductance (gs), intercellular CO_2_ concentration (Ci), and transpiration rate (Tr) in the four measurement periods. Periods I and II represent the relatively low and high soil water content in 2021, respectively. Periods III and IV represent the relatively low and high soil water content in 2022, respectively. Error bars represent standard errors (*n* = 3).

Diurnal courses of *PAR*, vapor pressure deficit, net photosynthetic rate, stomatal conductance, intercellular CO_2_ concentration, and transpiration rate for measurement periods are shown in Figure 2. The net photosynthetic rate and stomatal conductance of two plots reached their daily peak at 10:00. The transpiration rate in both plots reached their peaks at 10:00 in periods I and III, and the peaks were observed at 11:00 in period II. The values along diurnal courses of these three parameters in the control plot were constantly higher than those in the treated plot. In addition, these three photosynthetic parameters of two plots all showed a single peak pattern in 2021. The intercellular CO_2_ concentration peaked before sunrise, and its diurnal variations were opposite to stomatal conductance trends in both plots. The intercellular CO_2_ concentration of the control plot was generally higher than that of the treated plot in period I.

Notably, in 2022 (periods III and IV), net photosynthetic rate of the treated plot reached the peak at 10:00 and then decreased, while intercellular CO_2_ concentration increased, occurring at the midday depression of photosynthesis. Stomatal conductance and transpiration rate of the treated plot also showed the double peak pattern in 2022. Transpiration rate in the treated plot reached the peak at 10:00 in period IV, whereas the peak value in the control plot was observed at 11:00. Furthermore, the intercellular CO_2_ concentration in the treated plot rose at 11:00 and then exceeded that of the control plot.

**Figure 3:**
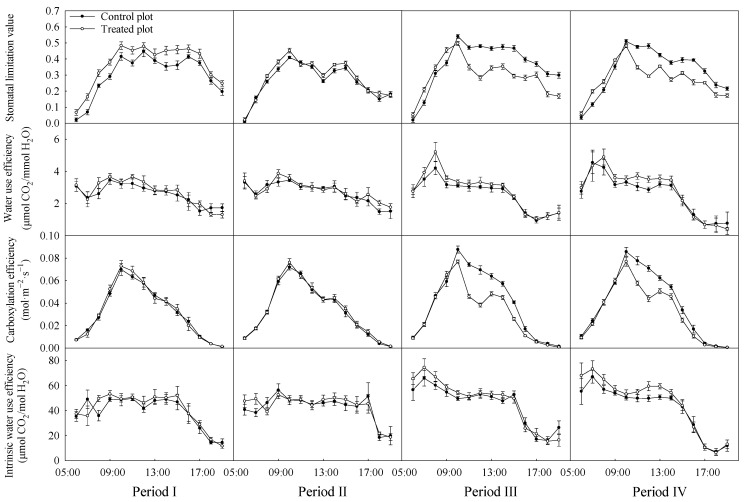
Diurnal courses of stomatal limitation value, water use efficiency, carboxylation efficiency and intrinsic water use efficiency in the four experimental periods. Periods I and II represent the relatively low and high soil water content in 2021, respectively. Periods III and IV represent the relatively low and high soil water content in 2022, respectively. Error bars represent standard errors (*n* = 3).

Figure 3 shows the diurnal changes of stomatal limitation value, water use efficiency, carboxylation efficiency, and intrinsic water use efficiency in the four periods. The stomatal limitation value generally reached a peak at around 10:00 and was higher in the treated plot than in the control plot in 2021. The stomatal limitation value of the treated plot decreased at 11:00 and then was constantly lower than that of the control plot in 2022, indicating that the intercellular CO_2_ concentration increased at this time. The carboxylation efficiency showed a single peak pattern in 2021 and showed a midday decline in 2022. The water use efficiency and intrinsic water use efficiency of the treated plot were generally higher than those of the control plot.

### 3.3. Differences in Daily Averages of the Photosynthetic Parameters

**Figure 4:**
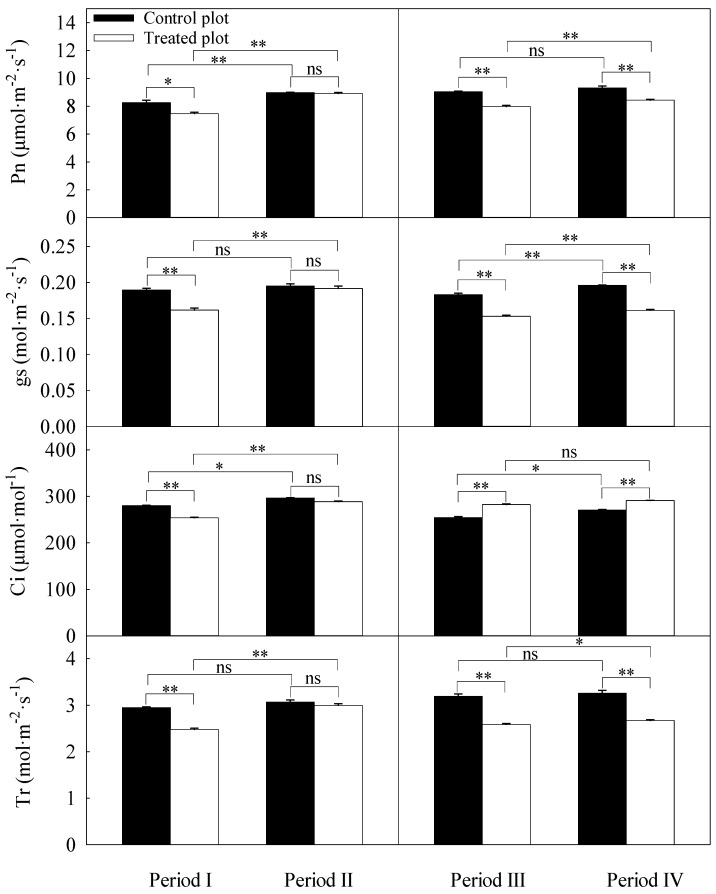
Daily averages of net photosynthetic rate (Pn), stomatal conductance (gs), intercellular CO_2_ concentration (Ci), and transpiration rate (Tr) during each period. Error bars represent standard errors (*n* = 3). Periods I and II represent the relatively low and high soil water content in 2021, respectively. Periods III and IV represent the relatively low and high soil water content in 2022, respectively. Significant differences were checked by *t*-test. *, significant difference at *p* ≤ 0.05. **, significant difference at *p* ≤ 0.01. ns, no significant difference.

The daily averages for the gas exchange parameters of the treated plot were significantly different from those of the control plot except for period II (Figure 4). The net photosynthetic rate of the treated plot was lower than that of the control plot in four periods. The stomatal conductance of the treated plot was 14.61, 16.31, and 17.71% lower than that of the control plot in periods I, III, and IV, respectively. The transpiration rate of the treated plot was 15.93, 18.89, and 18.11% lower than that of the control plot, respectively. These three photosynthetic parameters of the treated plot were significantly greater in the period of higher SWC in each year. Moreover, the intercellular CO_2_ concentration in the treated plot was significantly lower than that in the control plot in period I, whereas in period III and IV, the values were higher in the treated plot. The significant effect for treatment, period, and their interaction for each parameter is presented in Table 2. Except for the low effect of treatment on intercellular CO_2_ concentration, the effects for parameters were significant.

**Figure 5:**
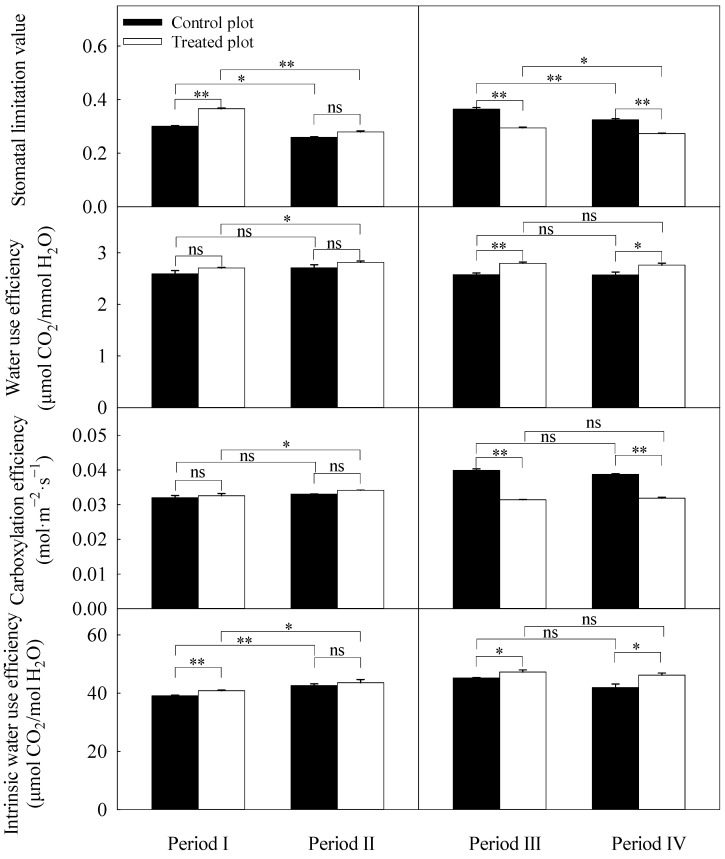
Daily averages of stomatal limitation value (Ls), water use efficiency (WUE), carboxylation efficiency (CE), and intrinsic water use efficiency (WUEi) in each experimental period. Periods I and II represent the relatively low and high soil water content in 2021, respectively. Periods III and IV represent the relatively low and high soil water content in 2022, respectively. Error bars represent standard errors (*n* = 3). Significant differences were checked by *t*-test. *, significant differences at *p* ≤ 0.05. **, significant differences at *p* ≤ 0.01. ns, no significant difference.

The daily averages of stomatal limitation value, water use efficiency, carboxylation efficiency, and intrinsic water use efficiency are shown in Figure 5. The stomatal limitation value of the treated plot was significantly higher than that of the control plot in period I. However, in 2022, stomatal limitation values of the treated plot were significantly lower than those of the control plot. Although there was no significant difference in water use efficiency between plots in 2021, that in the treated plot was higher than in the control plots. However, there were significant differences in water use efficiency between the two plots in 2022. The carboxylation efficiency of the treated plot was significantly lower than that of the control plot in 2022. The intrinsic water use efficiency was significantly different between the plots except for period II. In addition, the stomatal limitation values in both plots were significantly lower in periods with higher SWC within each year. The significant effect from interactions of treatment and period on each parameter is presented in Table 3. Only stomatal limitation value was not significantly affected by treatment. Other parameters, except water use efficiency, were significantly affected by the period and interactions.

### 3.4. Light Response Curve Derived Parameters

In our study, in order to elucidate the effect of soil moisture change on the light response curve, measurements were performed in four periods between the two plots. Based on the fitted curves, relevant parameters in each measurement period are shown in Table 4. There was no significant difference in photosynthetic light response parameters between the two plots only in period II. The quantum use efficiency, maximum net photosynthetic rate, and light saturation point of the treated plot were significantly lower, and light compensation point and dark respiration rate were significantly higher than those of the control plot except for period II. In addition, the light saturation point, quantum use efficiency, and maximum net photosynthetic rate of the treated plot were significantly higher in period II than in period I, and the dark respiration rate and light compensation point showed the opposite trend. All parameters of the light response curve of the control plot were significantly different between the two periods in 2022. However, in the treated plot, only the light saturation point significantly increased in period IV.

### 3.5. CO_2_ Response Curve Derived Parameters

We conducted a comparative study on the response of photosynthesis of black locust to CO_2_ concentrations in two periods between the two plots. The parameters derived from CO_2_ response curves are shown in Table 5. There were significant differences in related parameters between the two plots in each period (measured only in periods III and IV). The CO_2_ saturation point, maximum net photosynthetic rate, and carboxylation efficiency of the treated plot were significantly lower than those of the control plot, and day respiration rate and CO_2_ compensation point were significantly higher. Furthermore, a significant decline in day respiration rate and CO_2_ compensation point was observed in both plots during period IV.

## 4. Discussion

### 4.1. Changes in Photosynthetic Characteristics under Rainfall Exclusion Treatment

The net photosynthetic rate and stomatal conductance of treated sample trees decreased significantly compared with control samples except for period II. This result is consistent with the conclusion of Wang et al. [39] that black locust was more sensitive to drought than *Platycladus orientalis*, with rapidly closing partial stomata and photosynthesis weakening. Lower stomatal conductance may reduce transpiration rate, and trees actively regulate stomatal aperture for photosynthesis. Water stress caused by exclusion treatment reduced stomatal conductance of black locust, which affected intercellular CO_2_ concentration and consequently weakened photosynthesis. Liu et al. [40] also discovered that water stress caused by changing precipitation patterns had negative effects on net photosynthetic rate, tree height, and basal diameter growth of black locust. The significant decline in gas exchange parameters was observed in many species under water stress [41,42,43].

Previous studies have shown that black locust was relatively sensitive to temporary soil moisture changes by rainfall [33]. The fluctuation of soil water content caused by rainfall could significantly affect plant photosynthesis [44,45]. Our study also found that gas exchange parameters in the treated plot were significantly improved after rainfall (period II), and photosynthetic parameters were not significantly different between plots. Liu et al. [46] showed that increasing soil water content enhanced the net photosynthetic rate, stomatal conductance, and transpiration rate of black locust. Despite rainfall exclusion treatment, the photosynthetic capacity of the treated plot recovered, probably related to better soil moisture conditions. Thus, the photosynthetic performance of black locust exhibits a certain degree of resilience and adaptability to seasonal soil moisture changes.

However, net photosynthetic rate of the treated plot was significantly lower than that of the control plot in period IV. This result indicates that the photosynthetic performance of the treated samples had not fully recovered. Furthermore, net photosynthetic rate and stomatal conductance were significantly affected by the treatment, period, and their interactions. These phenomena illustrate that long-term throughfall exclusion treatment may cause irreversible damage to photosynthetic organs and affect the photosystem, which is more obvious when rainfall events were few in 2022. The photosynthetic capacity of the treated samples could not recover after seasonal drought due to the long-term rainfall reduction treatment. This result is consistent with the observation of Grzesiak et al. [47] that, in the recovery period after end of long-term drought, gas exchange parameters of treatment did not fully return to the control level. After re-watering, stomatal conductance did not fully recover from successive drought on black locust, which decreased transpiration and photosynthesis [48]. In addition, Duan et al. [49] showed that seasonal drought reduced leaves’ gas exchange of four evergreen and two deciduous trees, and the recovery of photosynthesis and stomatal conductance after re-watering differed among species. Hence, the physiological response and degree of recovery of plant photosynthesis to water stress not only discriminate between species, but also depend on the intensity, frequency, and duration of drought.

The diurnal variation of photosynthesis in the treated plot had a photosynthetic midday depression phenomenon in 2022 (Figure 2). Previous studies also found that species exhibited a midday depression in diurnal courses of photosynthesis and transpiration during water deficit [50,51,52]. The treated sample trees employed photosynthetic midday depression for coping with excessive solar radiation, temperature, and vapor pressure deficit during throughfall exclusion treatment. This phenomenon may be related to the mechanism by which photosynthetic properties of drought-avoiding and drought-tolerant species respond to water deficit.

Collectively, relatively low soil moisture content by rainfall reduction would inevitably cause less photosynthesis. In the short term, the photosynthetic performance recovered with the supplement of soil moisture by rainfall. With prolonged period and aggravated intensity of drought, photosynthetic midday depression occurred in the treated plot, inhibiting photosynthetic activity of mesophyll cells. Although sample trees of the treated plot were in a relatively high soil water content, photosynthetic capacity was not fully recovered, altering photosynthetic characteristics of black locust. The long-term drought caused by rainfall exclusion treatment failed to recover photosynthetic performance of the treated plot after seasonal drought. Hence, these photosynthetic characteristics of black locust, especially as precipitation patterns are altered, warrant attention in future studies.

### 4.2. Stomatal Limitation and Non-Stomatal Limitation of Black Locust

In order to avoid dehydration, most plants close stomata during drought, reducing stomatal conductance, gas exchange, and inhibiting photosynthesis [53,54]. In our study, the stomatal conductance of the treated plot was significantly lower than of the control plot in period I, while stomatal limitation value was the opposite. Therefore, the decrease in net photosynthetic rate of the treated plot was mainly caused by stomatal limitation. For instance, Fenta et al. [55] and Benesova et al. [56] reported that drought-sensitive species exhibited stomatal limitation and decline of photosynthetic efficiency in response to short-term drought. Pepe et al. [57] indicated that stomatal limitation under seasonal drought stress dominated the decrease in net photosynthetic rate of black locust. Another study also demonstrated that the stomatal factor is key to governing CO_2_ assimilation during water deficit [58,59].

Mechanisms underlying weak photosynthesis caused by water stress include not only stomatal limitation but also non-stomatal limitation. The dominant limiting factors of photosynthesis are related to the intensity and duration of drought [60,61]. Zhang et al. [62] and Gao et al. [42] observed that long-term water stress resulted in non-stomatal limitation of photosynthesis in the afternoon and increased the midday depression of photosynthesis. With the extension of drought, the decline of net photosynthetic rate was mainly driven by non-stomatal limiting factors [63]. In 2022, the intercellular CO_2_ concentration of the treated plot was significantly higher than that of the control plot, while stomatal limitation values were reversed. This indicates that the decline in photosynthesis of the treated plot was mainly dominated by non-stomatal limitation.

In 2021, photosynthesis of treated samples was controlled mainly by stomatal limitation, and photosynthetic capacity recovered after seasonal drought (period II). Yang et al. [61] also reported that some reductions in photosynthetic traits were associated with stomatal limitation, but all treated trees recovered to control levels after seasonal drought. However, non-stomatal limitation was pronounced during photosynthesis of the treated plot in 2022, and photosynthetic performance did not present full recovery after seasonal drought (period IV). This is probably because the soil water content of the treated plot may not have reached the critical threshold for non-stomatal limitation in 2021. However, the lower SWC of the treated plot reached this threshold in 5-year throughfall exclusion treatment (2022), suppressing the recovery of photosynthetic capacity and potentially damaging photosynthesis resilience to drought.

The long-term drought caused by rainfall reduction treatment inevitably negatively affected the activity of leaf photosynthetic organs. This may have contributed to the decline in photosynthesis of treated samples, with non-stomatal limitation occurring. The lower assimilation rate of the treated plot led to the increase in intercellular CO_2_ concentration, which in turn triggered a decrease in stomatal conductance. During seasonal drought, the decrease in stomatal conductance was the reason for the decrease in photosynthesis in the control plot, but it may be the result of the decrease in photosynthesis in the treated plot. Unlike the control plot, long-term drought may cause non-stomatal limitation in the treated plot when subjected to seasonal drought. The stomatal limitation value was also significantly affected by the period and interactions. Therefore, the reasons for decline of photosynthesis in the treated plot changed from stomatal limitation to non-stomatal limitation. Such limitation factors’ variation can adjust gas exchange and reflect the sensitivity of stomatal and photosynthetic apparatus to drought.

These phenomena illustrate that long-term rainfall exclusion treatment would weaken the sensitivity of black locust photosynthetic performance to soil moisture changes after seasonal drought. The reduction in photosynthesis by non-stomatal limitation would further negatively affect the productivity of black locust, given that the intensity and frequency of drought should increase with global climate change. Such knowledge is crucial for elucidating the adjustments mechanism of black locust photosynthesis to long-term drought from a synergistic whole-tree approach.

### 4.3. Effect of Rainfall Exclusion Treatment on Light Response Curve

Photosynthetic light response curve is a momentous part of forest photosynthesis research [64]. It describes the dynamic variation in net photosynthetic rate as a function of light intensity and reflects the photosynthetic potential, light energy utilization, and other characteristics of plants [65]. Our results are typical and consistent with previous research [66,67,68]. For instance, Zhang et al. [62] observed that prolonged water stress decreased light saturation point, maximum net photosynthetic rate, and quantum use efficiency. A study conducted in rainfall reduction treatment showed that drought reduced maximum net photosynthetic rate, affecting the potential maximum assimilation rate of species [69]. The lower light saturation point and quantum use efficiency of the treated plot revealed that sample trees had a weak ability to utilize poor light, narrowed adaptation range to solar radiation, and inhibited photosynthetic capacity. Gao et al. [42] found that long-term water stress impaired the sensitivity of photosynthesis in response to light, which led to higher light compensation point and dark respiration rate. The higher light compensation point of the treated plot suggests that samples required higher light intensity to start accumulating CO_2_ assimilation products.

There was no significant difference in light response curve parameters between the two plots after rainfall (period II). It may be that better soil moisture conditions contribute to the recovery of the sensitivity of black locust to light intensity. However, these parameters of the two plots were significantly different in period IV with higher soil water content. It elucidated that long-term throughfall exclusion treatment irreversibly weakened the sensitivity of black locust photosynthetic characteristics to soil moisture changes. The sensitivity of photosynthetic characteristics to light cannot be fully recovered after seasonal drought due to long-term drought. Ultimately, our results indicate that drought induced by long-term rainfall exclusion treatment weakened leaves’ adaptability and sensitivity to light and soil moisture fluctuation and reduced the efficiency of light transformation.

### 4.4. The Sensitivity of Photosynthesis to CO_2_ Concentration Was Affected by Rainfall Exclusion Treatment

The photosynthetic CO_2_ response curve provides vital information on the photosynthetic process [70]. This curve can reflect the quantitative relationship between plant photosynthetic rate and CO_2_ concentration [71,72]. The carboxylation efficiency was regarded as an assimilative capacity of plant responses to low CO_2_ concentration, and it was inhibited under drought [73]. The lower carboxylation efficiency and CO_2_ saturation point of the treated plot indicate that throughfall exclusion treatment weakened the ability of samples to utilize low CO_2_ concentration, narrowed the adaptation range to CO_2_ concentration, and could not maintain high photosynthetic rate under high CO_2_ concentration conditions. The higher CO_2_ compensation point was observed in the treated plot, which indicates that treated samples required higher CO_2_ concentration to initiate photosynthesis and organic matter accumulation than control samples. Moreover, the day respiration rate and CO_2_ compensation point of both plots increased significantly in period IV (after rainfall), probably because of higher soil water content. Liu et al. [74] also discovered that photosynthetic CO_2_ response curve parameters differed significantly before and after the rainfall pulse. In summary, throughfall exclusion treatment weakened the short-term sensitivity of black locust photosynthesis to CO_2_ concentration changes.

### 4.5. Drought Response Strategies of Black Locust under Rainfall Exclusion Treatment

Plants usually adapt to water stress by improving water use efficiency and closing partial stomata [2,50,75,76]. For example, pines improved water use efficiency and reduced transpiration to response to summer seasonal drought [77]. Four tree species exhibited an acclimation pattern to drought by an increase in intrinsic water use efficiency [60]. Black locust of treated plots has shown similar drought response characteristics. Long-term rainfall exclusion treatment reduced the photosynthetic activity of black locust mesophyll cells. During temporally seasonal drought, the assimilation rate was reduced, directly leading to accumulation of excessive CO_2_ between cells, which resulted in a sharp decline in stomatal conductance. The decrease in stomatal conductance was inevitably accompanied by the decrease in transpiration rate, which eventually led to the increase in water use efficiency and intrinsic water use efficiency.

In our study, black locust’s raising water use efficiency and intrinsic water use efficiency is considered as a long-term drought response strategy for photosynthesis by optimizing evaporative water loss. Nadal-Sala et al. [78] also recorded that black locust exhibited relatively high water use efficiency compared with *Alnus glutinosa* and *Fraxinus excelsior* during long-term drought. This may reflect the different synchronous responses of photosynthesis and transpiration to soil moisture changes, resulting in acclimation of the promoting of plant water use efficiency to long-term water stress. Unlike intrinsic water use efficiency, only treatment had an effect on water use efficiency, which may be related to fewer measurement periods. The higher water use efficiency and intrinsic water use efficiency of treated samples allowed an effective carbon assimilation by reducing stomatal conductance and water consumption. Consequently, under rainfall exclusion treatment, elevated water use efficiency and intrinsic water use efficiency proved to be a strategy for treated samples’ adaptation to long-term drought.

## 5. Conclusions

Our study observed that declined throughfall negatively affected the photosynthetic capacity and leaf gas exchange of black locust. The rainfall exclusion treatment resulted in less sensitivity of photosynthesis to light intensity and CO_2_ concentration. The photosynthetic performance of the treated plot could recover in period II, but could not fully recover in period IV. In addition, the photosynthetic midday depression appeared in the treated plot in 2022, and the dominant factors for decline in photosynthesis changed. Although weakened photosynthetic performance inevitably affected the productivity of black locust, this effect may be mitigated by reducing stomatal conductance, increasing water use efficiency and intrinsic water use efficiency. These results elucidated the response of photosynthetic properties of black locust to drought, indicating that its potential resilience to precipitation change and photosynthetic capacity recovery gradually weakened with the prolongation of drought. Long-term rainfall exclusion treatment affected the responses of photosynthetic characteristics to seasonal drought. In future studies, organs’ (leaf, roots, or shoots) water potential measurements will be added to better characterize tree water status and precisely define soil drought and the effects of drought on plantation water status. This has crucial implications for predicting sustainable development of vegetation and ecosystem function under global precipitation pattern changes.

## Figures and Tables

**Table 1:** Daily averaged soil water content (SWC, m^3^·m^−3^) in 1 m profile over two weeks before the measurement days and measurement period in each plot. Periods I and II represent the relatively low and high soil water content in 2021, respectively. Periods III and IV represent the relatively low and high soil water content in 2022, respectively. The values represent the mean ± SE (*n* = 336 before the measurement days, *n* = 72 measurement period).

Period	Before the Two Weeks SWC		Measurement Period SWC	
	Control Plot	Treated Plot	Control Plot	Treated Plot
Period I (28–30 July 2021)	0.267 ± 0.020	0.234 ± 0.033	0.268 ± 0.004	0.239 ± 0.006
Period II (9–11 September 2021)	0.265 ± 0.037	0.216 ± 0.035	0.284 ± 0.003	0.252 ± 0.004
Period III (23–25 June 2022)	0.265 ± 0.006	0.232 ± 0.004	0.255 ± 0.002	0.224 ± 0.001
Period IV (1–3 August 2022)	0.294 ± 0.005	0.243 ± 0.007	0.283 ± 0.002	0.244 ± 0.001

**Table 2:** *p* values of repeated measures ANOVA for the parameters in Figure 4 on the effects of the treatment, periods, and their interactions.

Source of Variation	df	Pn	gs	Ci	Tr
Treatment	1	0.001	<0.001	0.328	<0.001
Period	3	0.016	0.005	<0.001	0.015
Treatment × Period	3	0.014	0.005	0.001	0.012

Note: Pn: net photosynthetic rate (μmol·m^−2^·s^−1^); gs: stomatal conductance (mol m^−2^·s^−1^); Ci: intercellular CO_2_ concentration (μmol mol^−1^); Tr: transpiration rate (mmol·m^−2^·s^−1^).

**Table 3:** *p* values of repeated measures ANOVA for the parameters in Figure 5 on the effects of treatment, periods, and their interactions.

Source of Variation	df	Ls	WUE	CE	WUEi
Treatment	1	0.328	<0.001	<0.001	<0.001
Period	3	<0.001	0.341	0.046	0.046
Treatment × Period	3	0.001	0.597	0.021	0.021

Note: Ls: stomatal limitation value; WUE: water use efficiency (μmol CO_2_/mmol H_2_O); CE: carboxylation efficiency (mol·m^−2^·s^−1^); WUEi: intrinsic water use efficiency (μmol CO_2_/mol H_2_O).

**Table 4:** Parameters derived from light response curves in each period. Periods I and II represent the relatively low and high soil water content in 2021, respectively. Periods III and IV represent the relatively low and high soil water content in 2022, respectively. The values represent the mean ± SE (*n* = 3). Significant differences were checked by *t*-test. Different lowercase letters indicate significant differences at *p* ≤ 0.05 between the two plots for each period. Significant difference between the two periods for each plot is expressed by *p*-value.

Study Period	Plot	LSP	LCP	AQE	P_max_	R_d_
Period I	Control	1726.12 ± 35.20 a	21.58 ± 3.28 a	0.06 ± 0.003 a	16.89 ± 0.11 a	1.07 ± 0.19 a
	Treated	1663.20 ± 22.91 b	28.25 ± 2.78 b	0.05 ± 0.003 b	16.53 ± 0.14 b	1.40 ± 0.12 b
Period II	Control	1881.37 ± 107.30 a	17.21 ± 1.87 a	0.07 ± 0.003 a	16.98 ± 0.20 a	1.03 ± 0.10 a
	Treated	1823.86 ± 79.81 a	18.38 ± 2.43 a	0.07 ± 0.004 a	16.91 ± 0.11 a	1.15 ± 0.20 a
Periods I and II	*p*_Control_	0.022	0.032	0.001	0.414	0.685
Periods I and II	*p*_Treated_	0.006	0.001	0.001	0.001	0.044
Period III	Control	1707.53 ± 32.69 a	22.69 ± 0.79 a	0.06 ± 0.001 a	16.80 ± 0.03 a	1.27 ± 0.05 a
	Treated	1648.55 ± 19.14 b	31.70 ± 2.33 b	0.05 ± 0.003 b	16.37 ± 0.09 b	1.58 ± 0.05 b
Period IV	Control	1868.64 ± 24.25 a	18.30 ± 1.07 a	0.07 ± 0.002 a	17.02 ± 0.07 a	1.12 ± 0.04 a
	Treated	1751.47 ± 25.95 b	30.76 ± 0.79 b	0.05 ± 0.001 b	16.52 ± 0.07 b	1.44 ± 0.07 b
Periods III and IV	*p*_Control_	0.009	0.011	0.019	0.04	0.027
Periods III and IV	*p*_Treated_	0.011	0.633	0.384	0.155	0.09

Note: LSP: light saturation point (μmol·m^−2^·s^−1^); LCP: light compensation point (μmol·m^−2^·s^−1^); AQE: quantum use efficiency (μmol·μmol^−1^); P_max_: maximum net photosynthetic rate (μmol·m^−2^·s^−1^); R_d_: dark respiration rate (μmol·m^−2^·s^−1^).

**Table 5:** CO_2_ response curve of the net photosynthetic rate for two soil water contents. Periods I and II represent the relatively low and high soil water content in 2021, respectively. Periods III and IV represent the relatively low and high soil water content in 2022, respectively. The values represent the mean ± SE (*n* = 3). Significant differences were checked by *t*-test. Different lowercase letters indicate significant differences at *p* ≤ 0.05 between the two plots for each period. Significant difference between the two periods for each plot is expressed by *p*-value.

Study Period	Plot	CSP	CCP	R_p_	P_max_	CE
Period III	Control	1297.84 ± 27.18 a	60.35 ± 0.35 a	4.28 ± 0.02 a	27.15 ± 0.11 a	0.08 ± 0.0004 a
	Treated	1198.60 ± 10.36 b	66.33 ± 0.70 b	4.57 ± 0.07 b	26.31 ± 0.12 b	0.07 ± 0.0004 b
Period IV	Control	1255.56 ± 18.63 a	57.09 ± 0.43 a	4.09 ± 0.04 a	27.52 ± 0.39 a	0.08 ± 0.0001 a
	Treated	1198.90 ± 12.53 b	61.17 ± 0.44 b	4.28 ± 0.03 b	26.41 ± 0.26 b	0.07 ± 0.0002 b
Period III and IV	*p*_Control_	0.153	0.001	0.014	0.307	0.37
Period III and IV	*p*_Treated_	0.98	0.002	0.005	0.676	0.079

Note: CSP: CO_2_ saturation point (μmol·mol^−1^); CCP: CO_2_ compensation point (μmol·mol^−1^); R_p_: day respiration rate (μmol·m^−2^·s^−1^); P_max_: maximum net photosynthetic rate (μmol·m^−2^·s^−1^); CE: carboxylation efficiency (mol·m^−2^·s^−1^).

## Data Availability

Data are contained within the article.
